# Beyond benchmark accuracy: Toward trustworthy, open, and agentic clinical artificial intelligence

**DOI:** 10.1002/ped4.70062

**Published:** 2026-04-25

**Authors:** Partha Pratim Ray

**Affiliations:** ^1^ Department of Computer Applications Sikkim University Gangtok India

To the Editor:

The recent study on large language models (LLMs) for pediatric diagnosis presents compelling evidence that advanced models can surpass human clinicians in structured diagnostic tasks, particularly in rare disease identification.^1^ While these findings mark a significant milestone in clinical artificial intelligence (AI), they also necessitate a deeper reflection on how such systems should evolve for safe, scalable, and ethically grounded deployment in healthcare.

A central implication of the study lies not merely in performance gains, but in the demonstrated human–AI complementarity, where union accuracy significantly exceeds individual performance. This observation strongly advocates for a transition from standalone LLM usage toward agentic clinical systems, multi‐component architectures where LLMs interact with retrieval modules, verification engines, and structured medical knowledge bases. Such agentic approaches can enable iterative reasoning, contextual grounding, and dynamic hypothesis refinement, thereby addressing current limitations such as response inconsistency and overconfidence observed in reasoning‐optimized models.

In this context, the role of open‐source, fine‐tuned LLMs becomes particularly important.^2^, ^3^ While proprietary models dominate current benchmarks, open‐source alternatives‐when domain‐adapted using curated clinical datasets‐offer transparency, reproducibility, and the ability to embed institution‐specific knowledge. Fine‐tuned models aligned with clinical ontologies (e.g., Systematized Nomenclature of Medicine – Clinical Terms and Human Phenotype Ontology) can further enhance interpretability and reduce hallucination risks, especially in multilingual and resource‐constrained settings. Moreover, open ecosystems facilitate rigorous peer validation, which is essential for high‐risk applications such as medical diagnosis.

However, the ethical and safety dimensions remain critical. The study highlights variability in model outputs and the presence of potentially harmful diagnostic suggestions despite overall coherence. Such risks underscore the necessity of human‐in‐the‐loop oversight, calibrated uncertainty estimation, and abstention mechanisms when confidence is low.^4^ Additionally, concerns related to data privacy, bias in training corpora, and regulatory compliance, particularly under emerging frameworks like the EU AI Act, must be systematically addressed.^5^ Overreliance on LLMs may also contribute to diagnostic deskilling, necessitating careful integration strategies that preserve clinician autonomy and critical reasoning.

While current LLMs demonstrate remarkable diagnostic potential, their future lies not in isolated accuracy improvements but in open, verifiable, and agentic ecosystems that prioritize safety, transparency, and collaborative intelligence. Bridging this gap will be essential to transform LLMs from experimental tools into trustworthy clinical partners.

## CONFLICT OF INTEREST

The author declares no conflict of interest.
